# Proteome Differences in Placenta and Endometrium between Normal and Intrauterine Growth Restricted Pig Fetuses

**DOI:** 10.1371/journal.pone.0142396

**Published:** 2015-11-10

**Authors:** Fang Chen, Taiji Wang, Cuiping Feng, Gang Lin, Yuhua Zhu, Guoyao Wu, Gregory Johnson, Junjun Wang

**Affiliations:** 1 State Key Laboratory of Animal Nutrition, Alltech-MAFIC Research Alliance, College of Animal Science and Technology, China Agricultural University, Beijing, China; 2 Department of Animal Science, South China Agricultural University, Guangzhou, China; 3 Department of Obstetrics and Gynecology, China-Japan Friendship Hospital, Beijing, China; 4 Department of Animal Science, Texas A&M University, College Station, Texas, United States of America; 5 Department of Veterinary Integrative Biosciences, Texas A&M University, College Station, Texas, United States of America; University of Geneva, SWITZERLAND

## Abstract

Uteroplacental tissue plays a key role in substance exchanges between maternal and fetal circulation, and, therefore, in the growth and development of fetuses. In this study, proteomics and western blotting were applied to investigate the changes of proteome in the placenta and endometrium of normal and intrauterine growth restriction (IUGR) porcine fetuses during mid to late pregnancy (D60, 90, and 110 of gestation). Our results showed that proteins participating in cell structure, energy metabolism, stress response, cell turnover, as well as transport and metabolism of nutrients were differentially expressed in placenta and endometrium between normal and IUGR fetuses. Analysis of functions of these proteins suggests reductions in ATP production and nutrients transport, increases in oxidative stress and apoptosis, and impairment of cell metabolism in IUGR fetuses. Collectively, our findings aid in understanding of the mechanisms responsible for uteroplacental dysfunction in IUGR fetus, and are expected to provide new strategies to reduce fetal growth restriction in pigs and other mammals.

## Introduction

Intrauterine growth restriction (IUGR) commonly occurs in both human and livestock species [1] and impairs postnatal metabolism, growth, development and health [2]. In swine industry, IUGR is a major problem contributing to high neonatal morbidity and mortality, low efficiency of feed utilization and poor meat quality [3] and [4]. Therefore, it is of importance to investigate the underlying mechanisms behind IUGR and to find a solution to prevent its occurrence.

Placental transport of nutrients is a key determinant of fetal intrauterine growth [5]. The endometrium (a mucosal layer underlying placenta and a part of uterus) is also crucial for embryonic development, implantation, and placentation [6]. To provide an optimal environment for fetal growth, both placenta and endometrium undergo dramatic changes in morphology and function during pregnancy [7]. In pigs, placental development starts at approximately d 15 and reaches maximum by d 60–70 of gestation [8]. As a type of the epitheliochorial placenta, the pig trophoblast is attached to the uterine luminal epithelium without invasion during implantation, which is very different from implantation processes in humans. Over the mouths of uterine glands, pig allantochorion is not directly attached to the endometrial epithelium but forms areolae, where uterine gland secretions are absorbed by trophoblast cells. In the inter-areolar regions, substances pass from uterine circulation, via the uterine epithelium, into the trophoblast, and after the placenta is formed, via the capillary in fetal placenta into fetal circulation [9]. Although previous studies indicate that insufficiency in placental and endometrial development and function contributes to IUGR, little is known about changes of their proteomes at different gestational stages. The objective of this study was to identify differences in placental and endometrial proteins between normal body weight (NBW) and IUGR fetuses at d 60, 90, and 110 of gestation.

## Materials and Methods

This experiment was approved by China Agricultural University Animal Care and Use Committee (No. 20080106–1). All surgery was performed under anesthesia with halothane, and all efforts were made to minimize discomfort.

### Animal experiment and sample collection

Twenty-four gilts (Landrace × Large White) were used in this study. These gilts were mated, housed individually. They had free access to drinking water and were fed a corn-and soybean meal-based diet (2 kg.d^-1^) formulated to meet or exceed nutrient requirements according to the National Research Council (2012). At d 60, 90 and 110 of gestation, 8 gilts were randomly selected and exsanguinated after induction of anesthesia with halothane [10]. The maternal abdomen was opened and the pregnant uterus was extracted immediately from the body cavity. IUGR fetuses were identified as those whose weights were less than two standard deviation of the mean body weight for gestational age. One NBW fetus and one IUGR fetus, as well as their corresponding placenta and endometrium, were collected from each one of gilts. Placenta was easily separated from endometrium on d 60, 90 and 110 of gestation, and we did not observe any contamination between these two tissues. The tissues were rapidly transferred to liquid nitrogen and stored at -80°C.

### Extraction of proteins from placental and endometrial samples

The placenta and endometrium from 18 IUGR and 18 NBW (6/stage) were used to extract protein, as we described previously [3]. Briefly, approximately 0.2 g frozen samples were crushed to powder in liquid nitrogen, then homogenized in a lysis buffer containing 7 M urea, 2 M thiourea, 4% 3-[3-(-cholamidopropyl)-dimethylammonio]-1-propanesulfonate, and 50 mM dithiothreitol with protease inhibitors (GE Healthcare, Piscataway, NJ). An ultrasonicater (Sonics Model VC 750, Sonics and Materials, Newtown, CT) was set at 20% power output and used to break down the mixture for 10 min at 0°C. After the addition of 1% (vol/vol) nuclease mix (GE Healthcare), the mixture solution was kept at room temperature for 1 h to completely solubilize proteins, followed by re-sonification for 10 min as described above to thoroughly break down cell membranes. The homogenate was centrifuged for 10 min at 13,000 g at 4°C to settle down the insoluble components. The supernatant fluid was obtained and its protein concentration was determined using the Brandford method. Portions of the homogenate (1 mg of protein for both placenta and endometrium) were stored at -80°C.

### Comparative Proteome Analysis

Two-dimensional gel electrophoresis was run in triplicate for both placental and endometrial samples at each of 3 time-points (D60, 90, 110) to compare protein expression between IUGR and NBW fetuses. First, isoelectric focusing was performed using immobile DryStrip gels (pH 3 to 10, nonlinear, 24 cm long; GE Healthcare), as we described previously [11]. One mg protein was loaded onto immobile Drystrip gels strips using an in-gel sample rehydration technique. The first-dimensional isoelectric focusing was carried out at 20°C for 100,000 voltage h (IPGphor system, GE Healthcare), followed by equilibration in 4 mL of equilibration buffer 1 (6 M urea, 1% dithiothreitol, 30% glycerol, and 50 mM Tris-Cl pH 8.8) for 15 min on a shaker and then in 4 mL of equilibration buffer 2 (6 M urea, 2.5% iodoacetamide, 30% glycerol, 50 mM Tris-Cl pH 8.8) for 15 min as described above. The second-dimensional Gel was performed using 12.5% SDS-PAGE gels. The gels were run at 30 mA/gel for 30 min and then at 50 mA/gel until the Bromophenol Blue came out of the gels. Analytical gels were stained with Coomassie Brilliant Blue G-250 (Amresco, Solon, OH), and high-resolution gel images (400 dpi) were obtained using a scanner (ImageScanner, model PowerLook 2100XL, UMAX Technologies, Atlanta, GA). Images were analyzed using commercial software (Image-Master 2D Platinum Version 6.01, GE Healthcare) and differentially expressed protein spots that deviated more than 1.3-fold in their relative volume (% vol) were selected for in-gel digestion and protein identification.

### In-Gel Digestion and MALDI-TOF/TOF MS Analysis

Protein spots of interest were cut from the gels to be destained with 100 μL of 50% (v/v) acetonitrile (ACN) in 25 mM ammonium bicarbonate for 1 h. Then an aliquot (3 μL) of trypsin solution (10 μg/mL; Amresco Inc., Solon, OH) in 25 mM ammonium bicarbonate was added to each gel piece after it was completely dried by vacuum centrifugation (Eppendorf Concentrator 5301, Eppendorf, Hamburg, Germany) for 30 min. The mixture was incubated in a trypsin solution at 4°C for rehydration for 1 h, followed by incubation at 37°C for 12 h. Thereafter, the gel pieces were vacuum-dried to evaporate the solvent and 8 μL of 5% trifluoroacetic acid (TFA) was added onto the dry gel pieces. After incubation at 37°C for 1 h, the solution was transferred into a microcentrifuge tube. The gel pieces were extracted twice separately with 8 μL of 2.5% TFA and 50% acetonitrile, and 5% TFA. The third extraction was performed using 8 μL of 100% acetonitrile and the solution was combined with the previous two extracts in the microcentrifuge tube. Finally, the combined solution was dried and resolubilized in 2 μL of 0.5% TFA for protein identification using MALDI-TOF/TOF MS (Matrix Assisted Laser De sorption Ionization-Time of Flight/Time of Flight MS).

### Protein Identification

The peptide mass fingerprint analysis was performed using a search engine (MASCOT, Matrix Science, Lon- don, UK) [12]. Searching parameters included: (1) “trypsin” as the enzyme of protein digestion; (2) “monoisotopic” as mass value; (3) “unrestricted” as peptide mass; (4) “0.3 Da” as peptide mass tolerance; (5) “oxidation (M) and carbamidomethyl (C)” as variable modifications; and (6) “1” as maximum missed cleavages. Protein match with a score was considered significant (*P* < 0.05).

### Western Blotting

Extracted protein samples were boiled for 5 min and separated by electrophoresis (Bio-Rad, Richmond, CA) in 12% SDS-PAGE gel before electroblotted (Bio-Rad) onto a polyvinylidene fluoride membrane (Millipore, Billerica, MA) [11]. After polyvinylidene fluoride membranes were blotted with Tris buffer containing 5% fat-free dry milk and 0.05% Tween-20 (TBST; 0.05% Tween 20, 100 mmol/L of Tris-HCl, and 150 mmol/L of NaCl, pH 7.5) for 1 h at 25°C, they were rinsed in TBST for 4 times and incubated overnight at 4°C with primary antibodies, which were mouse polyclonal GAPDH (Abcom, ab22556), rabbit polyclonal HIF-1α (Abcam, ab114977), mouse monoclonal VEGF (Abcam, ab1316) and rabbit polyclonal VEGFR1 (Abcam, ab2350) with 1:3000, 1:2000, 1:2000 and 1:2000 dilutions, respectively. The membranes were washed in the same manner as described above and incubated with the horseradish peroxidase-conjugated secondary antibody (1:5,000; Sigma) for 40 min. Then, the membranes were washed 6 times for 5 min in TBST and the blots were detected using Lumi-light Western Blotting substrates (Biofuture, Beijing, China) and analyzed with the Imaging Analysis Software of National Institutes of Health (Bethesda, MD).

### Statistical analyses

Data are expressed as means ± SEM. Differences in fetal weights or western blot results in placenta or endometrium between IUGR and normal fetuses at a given day of gestation were statistically analyzed by the t-test (version 8.2; SAS Institute, Cary, NC, USA). Proteomic data for placenta or endometrium were analyzed by the t-test, a statistic program embedded in the commercial software (Image-Master 2D Platinum Version 6.01, GE Healthcare), to compare the difference between IUGR and normal fetuses at a given day of gestation. To avoid systemic error, we further calculated the ratio of protein levels between IUGR fetuses to those for normal weight fetuses, and only the proteins with a ratio higher than 1.3 or lower than -1.3 were chosen for further identification. *P* < 0.05 was taken to indicate statistical significance and data are expressed as means ± SEM.

## Results

### Body weight of fetuses

Both IUGR and NBW fetuses grew between D60 and D110 of gestation (Table 1). Body weights of selected IUGR fetuses were 93.4, 460, and 858 g respectively and were significantly lower than that of NBW at the same gestational day, which were 143.3, 914.3, and 1528 g respectively (*P* < 0.01, Table 1).

**Table 1:** Body weights of normal and IUGR fetuses at D 60, 90 and 110 of gestation.

Fetus	Body Weight^a^ (g)		
	D 60	D 90	D 110
**IUGR**	93.4 ± 8.5**^b^	460.0 ± 31.6**	858.3 ± 100.6**
**NBW**	143.3 ± 3.8	914.3 ± 70.56	1528.0 ± 77.6

^a^ Values are means ± SEM, n = 6 per group.

^b^ **, *P* < 0.01 vs the NBW group.

### Comparative proteome analysis of placentae between IUGR and NBW fetuses

**Fig 1:**
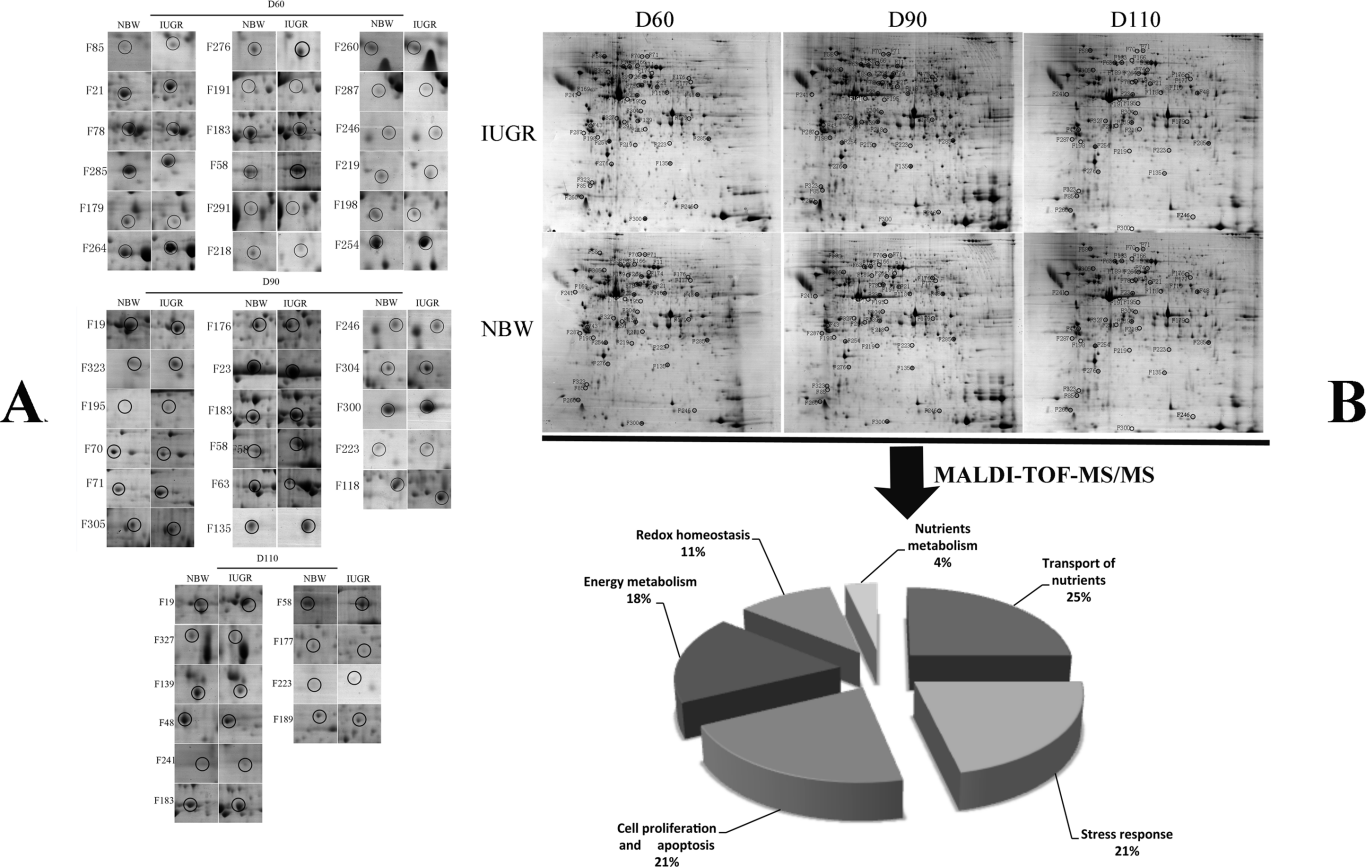
Abundance and classification of differentially expressed proteins between normal and IUGR placenta. (A) Abundance of differentially expressed proteins in the placenta of normal and IUGR pig fetuses at D 60, 90 and 110 during gestation. (B) Functional classification of differentially expressed proteins between placentae from normal and IUGR pig fetuses.

A total of 50 placental protein spots were differentially expressed in placentae between IUGR and NBW fetuses at D60, 90 and 110 of gestation. Their appearances in gel images were labeled in Fig 1A and biochemical information is summarized in Table 2 and Fig 1B. According to their biological function, these proteins were classified into the following groups: (1) energy metabolism; (2) nutrient transport; (3) stress response; (4) nutrient metabolism; (5) cell proliferation and apoptosis; (6) cell morphology and motility.

**Table 2:** Biochemical information about differentially expressed protein in the placenta of IUGR fetuses during mid and late gestation.

Spot No^a^	Protein name	Abb.	Access No	Score^b^	Gestation day	Ratio (I/N)^c^
**Energy metabolism**						
F285	Triosephosphate isomerase 1	TPI1	gi|61888856	176	60	-1.41
F176	Dihydrolipoamide dehydrogenase precursor	DLD	gi|47522940	117	90	-1.53
F48	Isocitrate dehydrogenase 1	IDH1	gi|89573979	143	110	-2.52
F179	Aconitase 1	ACO1	gi|115497728	322	60	-2.07
F23	Brain creatine kinase	CKB	gi|62751863	279	90	-2.00
**Transport of nutrients**						
F223	Alpha-fetoprotein precursor	AFP	gi|47523700	171	90	-10000
F223	Alpha-fetoprotein precursor	AFP	gi|47523700	190	110	-1.78
F189	Alpha-fetoprotein precursor	AFP	gi|47523700	200	110	-1.54
F219	Potassium voltage- gated channel	KCNH7	gi|194044147	131	60	-1.26
F118	Albumin	ALB	gi|833798	338	90	-1.34
F110	Albumin precursor	ALB	gi|30794280	256	110	-2.56
F198	Apolipoprotein A-I	APOA1	gi|164359	317	60	-1.72
F254	Apolipoprotein A-I	APOA1	gi|164359	317	60	-1.43
**Stress response**						
F264	Protein disulfide isomerase-associated 3 isoform 1	PDIA3I1	gi|194206717	208	60	10000
F276	Protein disulfide Isomerase-associated 3	PDIA3	gi|226875264	159	60	+1.71
F241	Chain A, Porcine Ribonuclease Inhibitor	RNH	gi|253723219	151	110	+1.78
F191	78 kDa glucose-regulated protein	GRP78	gi|66356310	195	60	+3.17
F183	75 kDa glucose-regulated protein	GRP75	gi|3122170	333	60	+2.05
F183	75 kDa glucose-regulated protein	GRP75	gi|3122170	333	90	+2.20
F183	75 kDa glucose-regulated protein	GRP75	gi|3122170	333	110	+3.50
F58	Heat shock 90kD protein 1, alpha	HSP90AA1	gi|60592792	200	60	+10000
F58	Heat shock 90kD protein 1, alpha	HSP90AA1	gi|60592792	200	90	+10000
F58	Heat shock 90kD protein 1, alpha	HSP90AA1	gi|60592792	200	110	+1.59
F63	Heat shock 71 kDa protein	HSPA8	gi|178847300	198	90	+1.52
F291	Heat shock 70kDa protein 5	HSPA5	gi|114626692	598	60	+3.85
F135	Parkinson disease protein 7	PARK7	gi|118403904	215	90	+1.46
**Nutrient metabolism**						
F218	Spermine synthase isoform 3	SPMSY	gi|149744293	111	60	1.39
**Cell proliferation and apoptosis**						
F260	Eukaryotic initiation factor 5A isoform I variant A	EIF5A2	gi|114666486	190	60	-1.89
F287	Eukaryotic translation initiation factor 6-like	EIF6	gi|109094726	135	60	-1.54
F287	Eukaryotic translation initiation factor 6-like	EIF6	gi|109094726	135	90	-1.83
F246	Aspartyl-tRNA synthetase, cytoplasmic	AspRs	gi|149730553	174	60	-4.80
F246	Aspartyl-tRNA synthetase	AspRs	gi|149730553	174	90	-1.40
F177	Zinc finger protein 420	ZFP420	gi|126329753	76	110	-1.69
F304	Cathepsin Z	CTSZ	gi|178057125	77	90	+1.39
F300	Cystatin-B	CSTB	gi|2494023	112	90	+1.31
**Cell morphology and motility**						
F19	ARP3 actin-related protein 3 homolog	ARP3	gi|23956222	141	90	+2.07
F19	ARP3 actin-related protein 3 homolog	ARP3	gi|23956222	141	110	+3.12
F172	Ezrin	EZR	gi|73945736	166	90	+2.60
F323	Myosin regulatory light chain	MRLC2	gi|149597555	152	90	+17.60
F85	Myosin regulatory light chain 12B isoform 2	MLC-2A	gi|114672411	598	60	+2.60
F195	Beta-actin	ACTB	gi|49868	137	90	+2.23
F164	Tubulin alpha-3	TUBA1B	gi|88683157	187	90	+1.74
F327	Annexin A8	Anx8	gi|194042330	243	110	-1.87
F139	Annexin A4	Anx4	gi|4033507	251	110	-1.30
F169	Moesin	MSN	gi|50513540	76	60	-1.70
F21	Keratin 7	KRT7	gi|114051856	450	60	-2.15
F78	Keratin 8	KRT8	gi|227430407	275	60	-1.78
F70	Gelsolin	GEL	gi|121118	197	90	-2.62
F71	Gelsolin	GEL	gi|121118	108	90	-4.86
F305	Tubulin alpha 1c	TUBAC	gi|221039556	127	90	-1.78

^a^Spot number refer to protein spot numbers that correspond to the labels in Fig 1.

^b^Protein score generated by MS-MS identification platform, with a score higher than 71 being considered as statistical significance.

^c^Ratio (I/N) refer to the ratio of protein levels in the endometrium of IUGR fetuses to these for normal-weight fetuses.

#### Energy metabolism

Five proteins involved in glucose and energy metabolism were expressed at lower levels in the IUGR placenta, as compared with the NBW placenta. These proteins included triosephosphate isomerase 1 (TPI1, Spot F285), dihydrolipoamide dehydrogenase precursor (DLD, Spot F176), isocitrate dehydrogenase 1 (IDH1, Spot F48), aconitase 1 (ACO1, F179), and creatine kinase (CKB, Spot F23).

#### Transport of nutrients

IUGR negatively affected the expression of several proteins involved in transport of nutrients, including alpha-fetoprotein precursor (AFP, Spot F223, F189), potassium voltage-gated channel subfamily H, member 7 (KCNH, Spot F219), albumin (ALB, Spot F118), albumin precursor (ALB, F110), and apolipoprotein A-I (APOA1, Spot F196, F254) (p < 0.01).

#### Stress response

Thirteen differentially expressed proteins that participate in stress response include: protein disulfide isomerase-associated 3 isoform 1 (PDIA3, Spot F264), protein disulfide isomerase-associated 3 (PDIA3, Spot 276) and chain A, porcine ribonuclese inhibitor (RNH, Spot F241), 78 kDa glucose-regulated protein (GRP78, Spot F191), 75 kDa glucose-regulated protein (GRP75, Spot183), heat shock 90 kD protein 1, alpha (HSP90A, F58), heat shock 71 kDa protein (HSPA8, Spot F63), heat shock 70 kDa protein 5 (HSPA5, Spot F291), and parkinson disease protein 7 (PARK7, Spot F135). All of these proteins were up regulated in the placenta of IUGR fetuses, as compared with NBW fetuses.

#### Nutrient metabolism

Spermine synthase isoform 3 (SPMSY, Spot F218), which is related to nutrient metabolism, was expressed at lower levels in the placenta of the IUGR placenta, compared with the NBW placenta.

#### Cell proliferation and apoptosis

Eight spots of proteins related to cell proliferation and apoptosis were affected by IUGR. Abundances of eukaryotic initiation factor 5A isoform I variant A (EIF5A2, Spot F260), eukaryotic translation initiation factor 6 (EIF6, Spot F287), aspartyl-tRNA synthetase (AspRs, Spot F174, F246) and zinc finger protein 420 (ZFP420, Spot F177) were lower in the IUGR placenta compared with the NBW placenta. In contrast, the levels of cystatin B (CSTB, Spot F300) and cathepsin Z (CTSZ, Spot F304) were higher in the IUGR group.

#### Cell morphology and motility

Fifteen protein spots related to cell morphology and motility were differentially expressed between IUGR and NBW placentae. Ezrin (EZR, Spot F172), myosin regulatory light chain (MRLC2, Spot F323), myosin regulatory light chain 12B isoform 2 (MLC-2A, Spot F85), beta-actin (ACTB, Spot F195), tublin alpha-3 (TUBA1B, Spot F164) and actin-related protein 3 (ARP3, Spot F19) were significantly up regulated in the IUGR placenta. In contrast, down regulation of annexin A4 (Anx4, Spot F327), annexin A8 (Anx8, Spot F139), moesin (MSN, Spot F169), keratin 7 (KRT7, Spot F21), keratin 8 (KRT8, Spot F78), gelsolin (GEL, Spot F70, F71) and tublin alpha 1c (TUBA1C) was detected in the IUGR fetuses.

### Comparative proteome analysis of endometria between normal and IUGR fetuses

**Fig 2:**
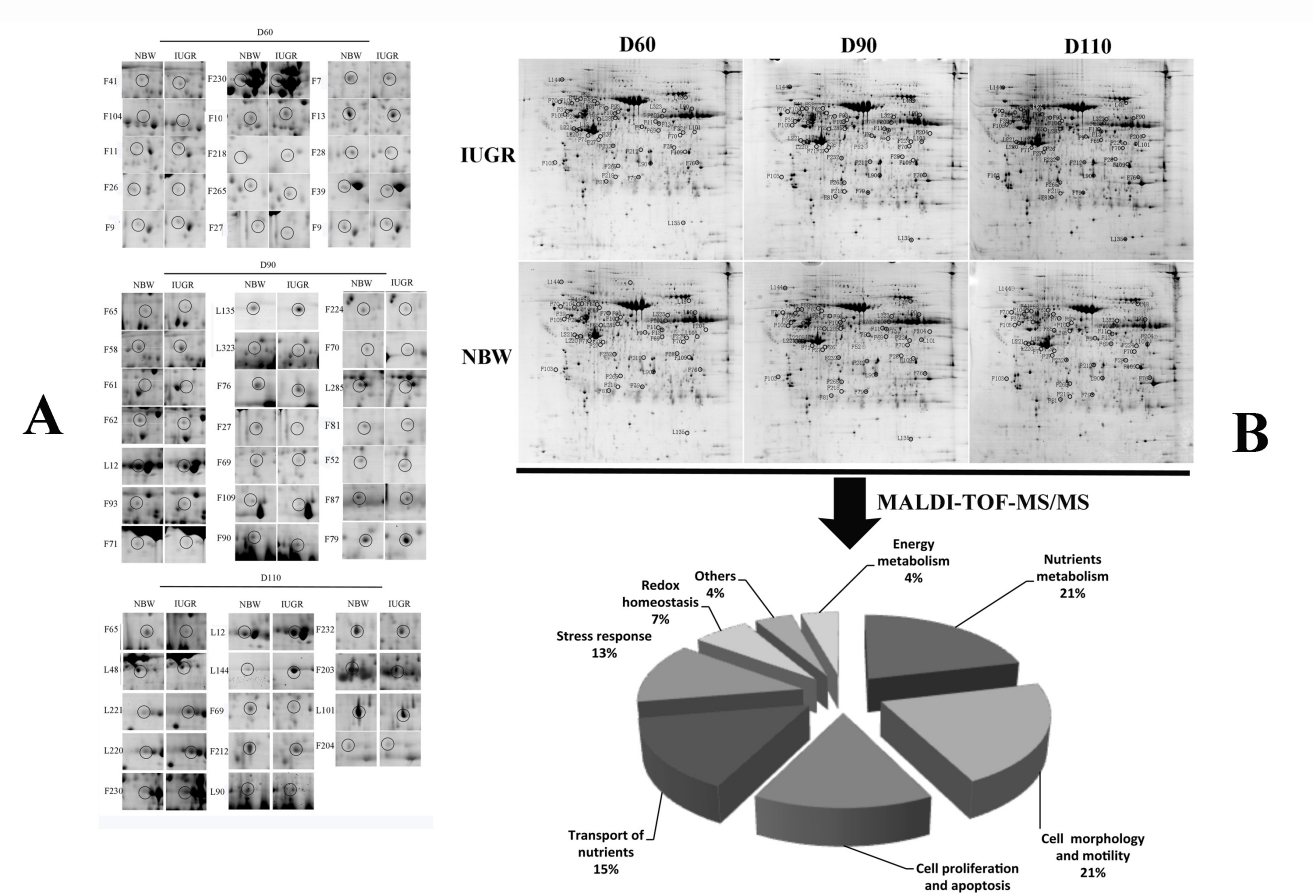
Abundance and classification of differentially expressed proteins between normal and IUGR endometrium. (A) Abundance of differentially expressed proteins in the endometrium of normal and IUGR pig fetuses at D 60, 90 and 110 of gestation. (B) Functional classification of differentially expressed proteins between endometrium from normal and IUGR pig fetuses.

A total of 52 protein spots were differentially expressed in the IUGR endometrium compared with NBW fetuses at D 60, 90 and 110 of gestation. Their appearances in gel images were labeled in Fig 2A and biochemical information is summarized in Table 3 and Fig 2B. According to the biological function, these proteins were involved in: (1) energy metabolism; (2) transportation; (3) stress response; (4) nutrient metabolism; (5) cell proliferation and apoptosis; (6) cell morphology and motility.

**Table 3:** Biochemical information about differentially expressed protein in the endometrium of IUGR fetuses during gestation.

Spot No^a^	Protein name	Abb.	Access No.	Score^b^		Gestation Day	Ratio (I/N)^c^
**Energy metabolism**							
F65	ATPase B subunit	V-ATPase	gi|162703	143	90		-2.28
F65	ATPase B subunit	V-ATPase	gi|162703	143	110		-2.97
F212	Pig Aldose Reductase Complexed With Idd384 Inhibitor	PAR	gi|8569627	113	110		-3.11
**Transport of nutrients**							
F27	Serum albumin	SALB	gi|73974957	82	60		-1.75
F27	Serum albumin	SALB	gi|73974957	82	90		-1.53
F69	Albumin	ALB	gi|833798	170	90		-10000
F69	Albumin	ALB	gi|833798	170	110		-1.90
F7	Albumin	ALB	gi|833798	79	60		-1.70
F232	Albumin	ALB	gi|833798	295	110		-1.85
F203	Albumin	ALB	gi|833798	140	110		-2.08
F109	Transferrin	TF	gi|136192	144	90		-1.88
F17	Copine-1	CPN1	gi|284795268	95	60		-1.70
**Stress response**							
F10	Thioredoxin domain containing 5	TXNDC5	gi|3035448	79	60		10000
F218	Peroxiredoxin-6	PRDX6	gi|47523870	103	60		+3.20
F79	Peroxiredoxin 6	PRDX6	gi|47523870	278	90		+2.90
L144	Glucose-regulated protein	GRP94	gi|255522883	276	110		+1.34
L135	Alpha-crystallin B chain	CRYAB	gi|75063982	124	90		+1.61
F215	Complement Component 3	C3	gi|47522844	82	110		-3.53
F265	Latexin	LXN	gi|194210342	119	60		-1.68
L323	Immunoglobulin gamma chain 6a	IgG	gi|210062464	110	90		-1.84
F76	Guanine nucleotide binding protein, beta polypeptide 2-like 1 isoform 1	GAQ	gi|73970309	158	90		-4.72
**Nutrient metabolism**							
F90	UDP-glucose dehydrogenase	UDPGD	gi|149702806	86	90		-2.63
F204	Argininosuccinate synthase	ASS1	gi|25453414	77	110		-1.59
F87	Arginine aminopeptidase	RNPEP	gi|21759001	148	90		-1.98
F224	Aspartate aminotransferase	AST	gi|47522636	97	90		-1.99
F70	Aspartate aminotransferase	AST	gi|47522636	84	90		-2.40
L285	Carnosinase 1	CNDP 1	gi|73945642	93	90		-1.86
L101	Cytosolic aspartate aminotransferase	AST	gi|47522636	335	110		-3.70
F81	Phosphoserine phosphatase	PSP	gi|262263179	172	90		-3.91
F13	Bifunctional 3'-phosphoadenosine 5'-phosphosulfate synthase 2-like	PAPSS2	gi|194042437	100	60		-1.52
**Cell proliferation and apoptosis**							
F58	Alpha-1-antichymotrypsin 2	ACT2	gi|194038345	268	90		1.84
F41	Alpha-1-Antichymotrypsin 3	ACT3	gi|194038347	181	60		1.91
F104	Alpha-1-antichymotrypsin 3	ACT3	gi|194038347	204	60		2.28
F104	Serpin A3-6	SERPINA3	gi|194038353	108	60		2.28
L48	Lamin-A/C	LMNA	gi|162139823	267	110		-1.76
F11	Elongation factor 1-gamma	eEF1A-1	gi|55977740	100	60		-1.32
F61	Heterogeneous nuclear ribonucleoprotein K	HNRNPK	gi|77736071	105	90		-1.98
F62	Heterogeneous nuclear ribonucleoprotein K	HNRNPK	gi|77736071	68	90		-4.93
**Cell morphology and motility**							
F26	Actin-related protein 3 isoform 2	ARP3	gi|73984196	108	60		-1.65
L221	Beta actin	ACTB	gi|15277503	100	110		+1.93
L220	Gamma-actin	ACTG	gi|109492380	85	110		+2.99
F9	T-complex protein 1 subunit beta	CCT2	gi|214010127	100	60		+1.79
F230	Tubulin, alpha, ubiquitous isoform 19	TBLA	gi|109096498	217	60		+1.39
F230	Tubulin, alpha, ubiquitous isoform 19	TBLA	gi|109096498	217	110		+2.19
F52	Actinin, alpha 4 isoform 12	ACTN4	gi|73947740	187	90		-2.18
L12	Cytokeratin 19	CK 19	gi|62751472	225	90		-3.22
L12	Cytokeratin 19	CK 19	gi|62751472	225	110		-1.28
F93	Cytokeratin 8	CK8	gi|227430407	98	90		-2.98
F71	Beta actin	ACTB	gi|15277503	137	90		-2.06
L90	Microtubule-actin crosslinking factor 1	Macf1	gi|123244271	93	110		-2.76
**Others**							
F28	Hypothetical protein PANDA_007173		gi|281338128	90	60		-2.14
F39	Protein of unknown function DUF1732		gi|289632714	82	60		-10000

^a^Spot number refer to protein spot numbers that correspond to the labels in Fig 2.

^b^Protein score generated by MS-MS identification platform, with a score higher than 71 being considered as statistical significance.

^c^Ratio (I/N) refer to the ratio of protein levels in the endometrium of IUGR fetuses to these for normal-weight fetuses.

#### Energy metabolism

Three proteins involved in glucose and energy metabolism exhibited differential expression in the endometria of IUGR and NBW fetuses. These proteins included H^+^-ATPase B subunit (V-ATPase, Spot F65, D90 and D110) and chain A, aldose reductase complexed with Idd384 inhibitor (Spot F212). Both proteins were expressed at lower levels in IUGR fetuses than in NBW fetuses.

#### Transport of nutrients

IUGR affected the expression of 9 proteins involved in the transport of nutrients. These proteins included albumin (Spot F27, F69, F7, F232, F203), transferrin (TF, Spot F109), and copine-1 (CPN1, Spot F7). All of these proteins were down regulated in the IUGR group, as compared with NBW fetuses.

#### Redox homeostasis

Three cellular redox homeostasis proteins were expressed at lower levels in the IUGR group. They were thioredoxin (TXN, Spot F10) and peroxiredoxin 6 (PRDX6, Spot F218, F79).

#### Stress response

Nine differentially expressed proteins play important roles in stress response. Compared with NBW fetuses, expression of thioredoxin (TXN, Spot F10), peroxiredoxin 6 (PRDX6, Spot F218, F79), glucose-regulated protein (GRP94, Spot L144) and alpha-crystallin B chain (CRYAB, Spot L135) was higher, while expression of latexin (LXN, Spot F265), complement component 3 (C3, Spot F215), immunoglobulin gamma (IgG, Spot L323), and guanine nucleotide binding protein (GAQ, Spot F76) was lower in the IUGR endometrium, as compared with the NBW group.

#### Nutrient metabolism

Nine protein spots related to nutrients metabolism were differentially expressed in the endometria between IUGR and NBW fetuses. These proteins included argininosuccinate synthase (ASS1, Spot F204), arginine aminopeptidase (RNPEP, Spot F87), aspartate aminotransferase (AST, Spots F224, F70, L101), carnosinase (CNDP1, L285), phosphoserine phosphatase (PSP, Spots F81), bifunctional 3'-phosphoadenosine 5'-phosphosulfate synthase 2-like (PAPSS2, Spot F13) and UDP-glucose dehydrogenase (UDPGD, Spot F90). The expression of these proteins was reduced in the IUGR endometrium, as compared with the NBW group.

#### Cell proliferation and apoptosis

Eight spots of proteins involved in cell proliferation and apoptosis were differentially expressed in the endometria of normal and IUGR fetuses. Abundances of alpha-1-antichymotrypsin 2 (ACT2, Spot F58), alpha-1-antichymotrypsin 3 (ACT3, Spot F41, F104) and serpin A3-6 (Spot F104) were increased in the IUGR group, as compared with the NBW group. In contrast, expression of lamin-A/C (LMNA, Spot L48), elongation factor 1 (eEF1A-1, Spot F11) and heterogeneous nuclear ribonucleoprotein K (HNRNPK, Spot F61, F62) was reduced in the IUGR endometrium, compared with the NBW group.

#### Cell morphology and motility

Twelve endometrial protein spots, which participate in cell structure and motility, were differentially expressed between IUGR and NBW fetuses. Actin-related protein 3 (Spot F26), actinin, alpha 4 isoform 12 (ACTN4, Spot F52), cytokeratin 19 (CK19, Spot L12), cytokeratin 8 (CK8, Spot F93), beta actin (ACTB, Spot F71), and microtubule-actin (Macf1, Spot L90), were down-regulated in the IUGR group, while T-complex protein 1 subunit (TCP1, Spot F9), tubulin, alpha, ubiquitous isoform 19 (TBLA, Spot F230), beta actin (ACTB, Spot L221), and gamma-actin (ACTG, Spot L220) were up-regulated in the IUGR group, as compared with NBW fetuses.

### Western blotting Analysis

**Fig 3:**
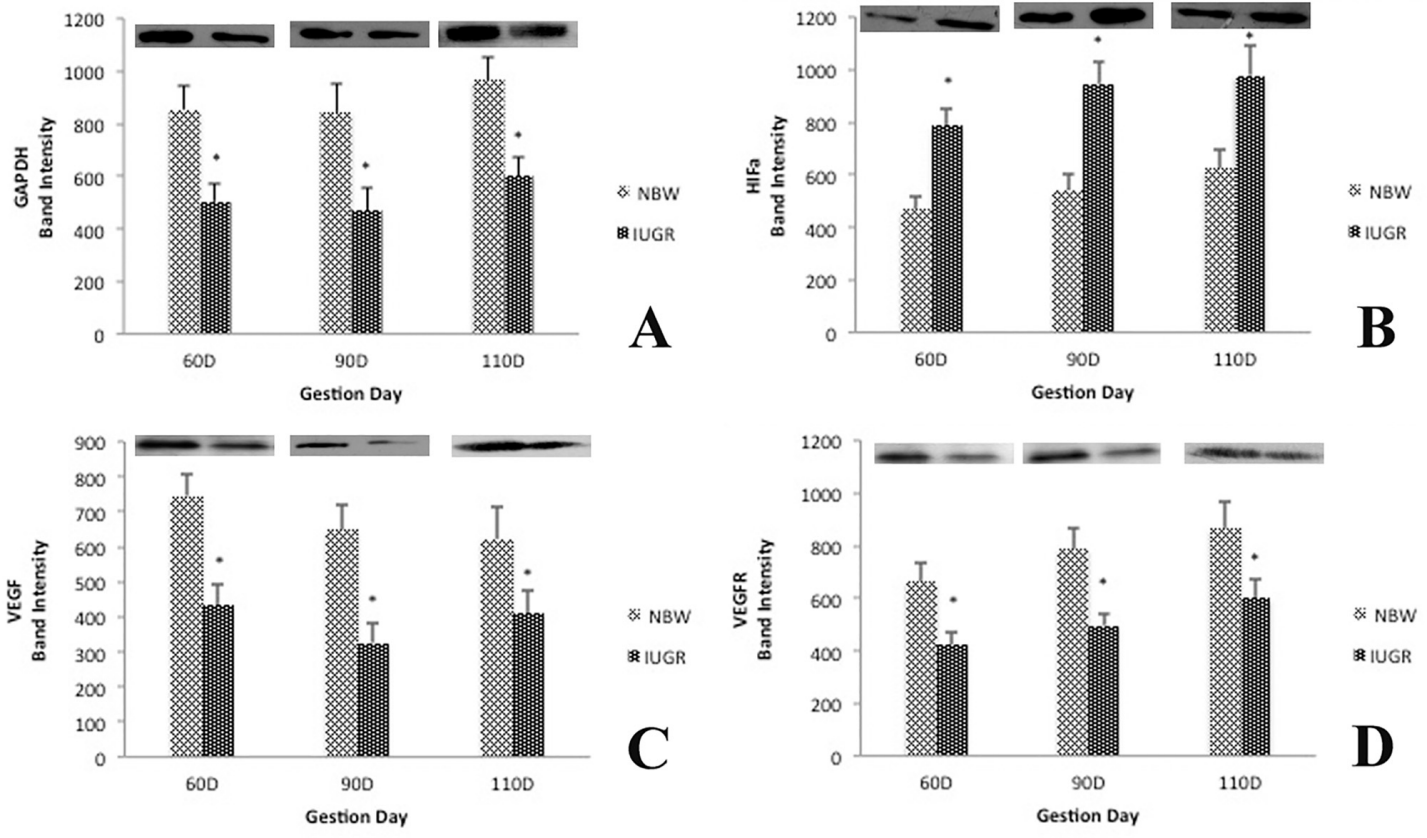
Expression of GAPDH, HIF-α, VEGF and VEGFR1 in placenta of normal and IUGR fetuses. (A) Western blot analysis of GAPDH. (B) Western blot analysis of HIF-α. (C) Western blot analysis of VEGF. (D) Western blot analysis of VEGFR1.

Fig 3 shows the western blotting analysis of GADPH, HIF-1α, VEGF and VREFR1. The expression of HIF-1α was higher in the IUGR placenta, compared with the NBW group. In contrast, GADPH, VEGF and VEGFR were reduced significantly (p < 0.01) in the IUGR placenta, as compared with the NBW fetuses.

## Discussion

IUGR is defined as impaired growth and development of the mammalian embryo/fetus or its organs during pregnancy and which is measured as fetal weight less than two standard deviation of the mean body weight for gestational age. The pigs possess noninvasive, diffuse type of epitheliochorial placentation. Sufficient development of both placenta and endometrium are of importance for substance exchange between maternal and fetal circulation, and, therefore, plays a key role in fetal growth and development. Our previous study indicates that plasma nutrient levels in umbilical vein differ between IUGR and normal fetuses [13], suggesting that nutrient transport by the uteroplacental unit is altered in the IUGR fetus. Therefore, we conducted the present study to investigate the changes of proteins in the placenta and endometrium between normal and IUGR pig fetuses at D60, 90, and 110 of gestation. Specifically, IUGR affected the expression of proteins involved in the following pathways: (1) energy metabolism; (2) transport of nutrients; (3) stress response; (4) nutrients metabolism; (5) cell proliferation and apoptosis; (6) cell morphology and motility.

### Energy metabolism

Energy is required for nutrient and gas transport between mother and fetus in the uteroplacental unit, as well as for the synthesis of hormones and secretion of molecules within trophoblasts [14]. An interesting finding of this study is that the expression of several key enzymes involved in energy metabolism was reduced in IUGR fetuses. TPI1, which catalyzes the reversible inter-conversion of the triose phosphate isomers dihydroxyacetone phosphate and D-glyceraldehyde 3-phosphate, plays an important role in glycolysis, and is essential for efficient ATP production. The reduced expression of TPI1 may provide less energy produced through glycolysis in the IUGR placenta, which is consistent with a 28% decrease in lactate production by the IUGR placenta [15]. In order to further confirm the impairment of glycolysis in the IUGR placenta, we analyzed expression GAPDH, a key regulatory protein, using the western blot technique, and the result was consistent with our proteomic analysis. DLD is a component of the pyruvate dehydrogenase complex and responsible for converting pyruvate into acetyl-CoA, which is then fed into the citric acid cycle and contributes to ATP production. IDH1 and ACO1 are also important enzymes participating in the citric acid cycle, an important metabolic pathway to produce ATP. The decreased expression of TPI, DLD and IDH in the IUGE placenta suggests less ATP production through the citric acid cycle, thereby compromising energy-dependent nutrient transport from mother to fetus. In addition, BCK and V-ATPases, both of which are related to ATP production, were reduced in the placenta and endometrium of IUGR fetuses, further confirming the alteration of energy metabolism in uteroplacental tissues of IUGR pig fetuses.

### Stress response

The uteroplacental tissues appear particularly vulnerable to oxidative stress due to extensive cell proliferation and high metabolic activity during pregnancy [16] and [17]. PARK7, which acts as a redox-sensitive chaperone and apparently protects cells against oxidative stress and apoptosis, is a sensor for oxidative stress. In this study, significant up-regulation of PARK7 expression provides evidence for the elevated levels of oxidative stress in the IUGR endometrium. Besides, abundances of several other proteins (PDIA3I1, PDIA3, TXN and PRDX6) related to cellular redox homeostasis were increased in the IUGR fetuses. PDIA3I1 and PDIA3 belong to the disulfide isomerase (PDI) family of proteins, which catalyze the reduction of mispaired thiol residues. Similarly, TXN1 (as an antioxidant by exchanging thiol-disulfide) can reduce placental oxidative stress and enhance fetal growth [18]. Furthermore, PRDX6 can protect the cell against oxidative stress or apoptosis by eliminating hydrogen peroxide (H_2_O_2_) and neutralizing other reactive oxygen species (ROS) [19]. The increased expression of PDIA3I, PDIA3, TXN1 and PRDX6 may suggest an adaptive protection against oxidative stress in IUGR utreoplacental tissues to compensate for fetal growth.

Hsps are molecular chaperones that control protein folding and protect cells and tissues from being damaged in adverse environmental conditions. Previous studies have indicated that Hsp70s and Hsp90s are strongly up-regulated by heat stress, oxidative stress, and toxic chemicals [20] and [21]. At the same time, expression of heat shock protein 70 was higher in placental tissues associated with increased apoptosis and decreased numbers of mitotic cells in cases of miscarriage [22]. In this study, expression of 5 members of heat shock 70 proteins family (GRP78, GRP75, Heat shock 71 kDa protein, HSPA8 and GRP94), HSP90A and CRYAB (a small heat shock protein) was all increased in the IUGR utero-placenta unit compared with the NBW counterpart, implying that uteroplacental tissues are under more intensive adverse stresses in IUGR fetuses, which may result in their poor development and organ dysfunction. Hypoxia is a major stress factor for the placenta during pregnancy. Therefore, we adopted the western blotting technique to confirm the increased expression of HIF-1α indicated from the proteomics analysis. Our results further support an augmented level of the HIF-1α protein in the IUGR placenta, as compared with the NBW group.

C3 plays a central role in activation of the complement system [23], contributing to innate immunity, and its deficiency can increase susceptibility to bacterial infection [24] and [25]. C3 is synthesized and secreted in the endometrium [26] and is expressed at a reduced level in patients with severe endometriosis [27]. Results of our study provide the first evidence that decreased abundance of C3 is associated with the IUGR endometrium, suggesting weaker immunity against infection in IUGR fetuses. This corroborates less abundance of IgG in the IUGR circulation.

### Transport of nutrients

Efficiency of substrate transport in uteroplacental tissues is crucial for fetal intrauterine growth by supplying nutrients from mother to fetus and by eliminating fetal metabolic wastes [28]. Previous studies have shown that the expression and activity of transport systems for glucose, amino acids and fatty acids were altered in the IUGR placenta [29] and [30]. In this study, we did not find any differences in expression of transporters for glucose, amino acids and fatty acids in the IUGR uteroplacental tissue, probably because their abundances were too low to be detected in the 2D gel image. However, we did identify that TF, which is responsible for iron binding and transport, was down regulated in the IUGR fetus. Maternal iron deficiency during pregnancy directly impairs embryonic growth and development [31], and strongly increases the risk for low birth weight and preterm delivery, as well as abnormal development of affected offspring [32]. Similarly, we previously reported a continuous reduction in the expression of TF in the jejunal mucosa of postnatal IUGR piglets between D 1 and D 21 after birth [33], which can now be explained by less iron transfer from mother to fetus during pregnancy.

Another important finding of this study is that abundances of proteins related to substrate transport, including ALB, AFP and APOA1, were down regulated in IUGR fetuses. ALB acts as a carrier of various substances, including lipid soluble molecules and certain minerals (e.g., steroid hormones, bile salts, unconjugated bilirubin, free fatty acids, calcium, and iron), in the blood circulation. ALB present in placental and endometrial tissues is derived from fetal and maternal circulation, respectively. AFP, produced by the yolk sac and the liver during fetal development, is considered to be the fetal form of ALB. The decreased levels of ALB and AFB in uteroplacental tissues may reduce substance exchanges between maternal and fetal circulation, contributing to the impairment of nutrient uptake and waste elimination in IUGR fetuses. APOA1, a major component of high-density lipoproteins, mainly participates in reverse transport of cholesterol from tissues back to the liver for excretion and plays an important role in cholesterol efflux from tissues. The decreased expression of APOA1 probably can result in higher levels of cholesterol in the IUGR placenta, which can result in more extensive stress in IUGR fetuses.

### Nutrient metabolism

Blood flow is a major determinant of uteoplacental function and fetal growth. Angiogenesis in the placental vasculature during pregnancy is necessary for increasing uteroplacental blood flow and, therefore, the supply of nutrients from maternal to fetal circulation [34]. Several previous studies reported that spermine plays an important role in angiogenesis, early mammalian embryogenesis, placental trophoblast growth, and embryonic development in the uterus [35] and [36]. In the current study, we found that spermine synthase was down regulated in the IUGR placenta, which could reduce the production of spermine and then placental vascular development. Consistent with this notion, results of the western blot analysis indicated that the expression of VEGF and VEGFR1 was decreased in the IUGR placenta, further contributing to compromised angiogenesis in the IUGR placenta.

The altered expression of several proteins related to amino acid metabolism in the IUGR endometrium could further aid in explanation of our previous findings of decreased concentrations of amino acids in the IUGR umbilical cord [13]. ASS1 is a key enzyme that catalyzes the synthesis of argininosuccinic acid (the immediate precursor of arginine) from citrulline and aspartic acid, and the loss of this enzyme is considered as a biomarker for susceptibility to arginine depletion [37]. Arginine is now known to be a nutritionally essential amino acid for mammalian fetal growth. RNPEP selectively catalyzes the cleavage of arginine and lysine from amino terminus of protein or peptide. The depressed expression of ASS1 and RNPEP may explain, in part, lower concentrations of arginine in the umbilical vein of IUGR fetuses. In addition, the expression of phosphoserine phosphatase, a key enzyme to produce serine, was up-regulated in the IUGR endometrium and could result in higher levels of serine in the fetal circulation, which is consistent with our previous results [13].

### Proliferation and apoptosis

To fulfill the metabolic demands of the growing fetuses, placenta and endometrium undergo dramatic morphological and histological changes during pregnancy, including active cell proliferation and extensive cell differentiation [38]. Stresses induced by maternal nutrient restriction, hypoxia and bacterial infection could adversely affect the growth and morphology of the placenta, causing reduction in the body weight of the fetus [39]. In this study, we find that expression of several key regulators for cell proliferation, including EIF5A2, EIF6, AspRs, and ZFP 420 [40, 41], were decreased in the IUGR placenta, and eEF1A-1, HNRNPK, and LAMN were decreased in the IUGR endometrium. The reduced expression of these proliferation-related proteins suggest slower rates of cell proliferation, which cannot meet the requirement for rapid placental development and lead to a smaller size of the placenta in IUGR fetuses.

Programmed cell death is a fundamental process for maintaining homeostasis in multi-cellular organisms and involves highly regulated processes, including pro-apoptotic and anti-apoptotic signal pathways. CTSZ is a lysosomal cysteine protease to hydrolyze proteins and have a vital role in mammalian cellular apoptosis, while CTSB is thought to play a key role in protecting cells against leaking of proteases from lysosomes during apoptosis. Enhanced expression of proteinase CTSZ, may imply higher apoptosis levels in the IUGR placenta, whereas enhanced expression of protease inhibitor CSTB may be related to protective reactions against apoptosis. In the endometrium, we found that 3 members of serpins (ACT2, ACT3 and serpin A3-6), all of which are capable of inhibiting proteases to control proteolytic cascades and mediate apoptosis processes as caspase inhibitors by preventing apoptosis in mammalian cells [42, 43], were increased in the IUGR group, implying more intensive apoptosis stress in the IUGR placenta.

### Cell morphology and motility

Fourteen proteins related to cell morphology and twelve proteins related to cell motility were differentially expressed in placentae and endometria between IUGR and NBW fetuses. Actin is a globular multi-functional protein that forms microfilaments of cytoskeleton and is essential for maintenance of cell shape and mobility. Several isoforms of actin were altered in IUGR, including ACTB (down-regulated in the IUGR placenta and endometrium); ACTN4, Macf1 (down-regulated in the IUGR endometrium); and ACTG (up-regulated in the IUGR endometrium). Besides, EZR, MRLC2, MLC-2A, MSN, GEL, and CCT2, which interact with actin to regulate cellular morphology, were differentially expressed between IUGR and NBW placentae. Similar results were obtained for ARP3 in the endometrium. In addition, both TUBA1B and TUBA1C, which are cytoskeleton microtubule elements, were differentially expressed in the placenta and endometrium. Furthermore, KRT7 and KRT8 were up regulated in the IUGR placenta, whereas CK19 was increased at D90, but deceased at D110 of gestation. Altered expression of these proteins related to actin, tublin and keratin could result in an abnormal change of cytoskeleton in the placenta and endometrium of IUGR fetuses.

Annexins provide a membrane scaffold and link to fibrinolysis, coagulation, inflammation and apoptosis. Reductions in two members of annexins (ANX4 and ANX8) may result in placental structural abnormality and dysfunction in IUGR fetuses. This novel finding provides another line of evidence for impaired utero-placental development in the IUGR conceptus.

## Conclusion

All the nutrients provided to fetuses during pregnancy are transported from maternal circulation through uteroplacenta tissue. Therefore, sufficient development of both placenta and endometrium (tissue size, vasculature, metabolism, secretory function) is very important for substance exchange between maternal and fetal circulation, and plays a key role in fetal growth and development. In order to fulfil substance transport between mother and fetus, the utero-placenta needs (1) energy and transporters; (2) an established vascular system. Results of the current work reveal that expression of several proteins related to energy metabolism, transportation, and vasculargenesis was reduced in IUGR placenta and endometrium, which could lead to inadequate energy provision and insufficient nutrient transport. Such utero-placental dysfunction could be a factor contributing to IUGR. Additionally, based on our proteomics data, we suggest that increased oxidative stress and apoptosis in IUGR placenta and endometrium, which can compromise utero-placental function, is another factor for the development of IUGR. Collectively, these findings have important implications for both human health and animal production to reduce the occurrence of IUGR fetuses.

## Supporting Information

S1 File ARRIVE Guidelines Checklist. (PDF)

S2 File Fetal Information for Gilts at D60, 90 and 110 of Gestation. (DOCX)
