# Supplementary material for: Proteome Differences in Placenta and Endometrium between Normal and Intrauterine Growth Restricted Pig Fetuses
Source: PLoS One. 2015 Nov 10;10(11):e0142396. doi: 10.1371/journal.pone.0142396 (PMC4640832; doi:10.1371/journal.pone.0142396)
Supplement: S2 File — (DOCX) [file pone.0142396.s002.docx]

**S1 Table. Fetal information for gilts at D60 of gestation.**

|  | **No. of Gilt** | | | | | | | |
| --- | --- | --- | --- | --- | --- | --- | --- | --- |
|  | **1** | **2** | **3** | **4** | **5** | **6** | **7** | **8** |
| **Fetal Number** | 14 | 13 | 11 | 16 | 15 | 13 | 12 | 10 |
| **IUGR Number** | 2 | 2 | 1 | 3 | 3 | 3 | 2 | 0 |
| **Average Body Weight** | 154 | 159 | 186 | 124 | 117 | 139 | 145 | 168 |

**S2 Table. Fetal information for gilts at D90 of gestation.**

|  | **No. of Gilt** | | | | | | | |
| --- | --- | --- | --- | --- | --- | --- | --- | --- |
|  | **9** | **10** | **11** | **12** | **13** | **14** | **15** | **16** |
| **Fetal Number** | 9 | 13 | 16 | 9 | 11 | 10 | 12 | 11 |
| **IUGR Number** | 1 | 3 | 3 | 2 | 2 | 3 | 2 | 3 |
| **Average Body Weight** | 875 | 729 | 567 | 790 | 818 | 568 | 753 | 673 |

**S3 Table. Fetal information for gilts at D110 of gestation.**

|  | **No. of Gilt** | | | | | | | |
| --- | --- | --- | --- | --- | --- | --- | --- | --- |
|  | **17** | **18** | **19** | **20** | **21** | **22** | **23** | **24** |
| **Fetal Number** | 11 | 10 | 10 | 12 | 10 | 12 | 9 | 11 |
| **IUGR Number** | 0 | 2 | 2 | 1 | 3 | 2 | 3 | 1 |
| **Average Body Weight** | 1445 | 1283 | 1270 | 1325 | 1232 | 1364 | 1211 | 1413 |
